# Morphometric Analysis of Petrous Bone With Emphasis on Landmarks Utilized in Lateral Skull Base Approaches

**DOI:** 10.7759/cureus.71760

**Published:** 2024-10-18

**Authors:** Anjulata Rai, Bidyarani L, Sachin Mittal, Yogesh Tyagi, Jyoti Arora, Jivak Bansal

**Affiliations:** 1 Anatomy, Atal Bihari Vajpayee Institute of Medical Sciences and Dr. Ram Manohar Lohia Hospital, New Delhi, IND; 2 Forensic Medicine and Toxicology, Atal Bihari Vajpayee Institute of Medical Sciences and Dr. Ram Manohar Lohia Hospital, New Delhi, IND; 3 Forensic Medicine and Toxicology, Vardhman Mahavir Medical College and Safdarjung Hospital, New Delhi, IND

**Keywords:** middle cranial fossa, morphometry, petrous bone, surgical anatomy, trigeminal ganglion

## Abstract

Background

The petrous part of the temporal bone plays a crucial role in various cranial surgical approaches, particularly those involving the middle cranial fossa. Understanding the morphometry of this region is essential for minimizing intraoperative risks and enhancing surgical outcomes. This study aims to provide a detailed morphometric analysis of the petrous bone and its anatomical landmarks in an Indian population, addressing a gap in the literature.

Methods

The study was conducted in the Department of Anatomy and Forensic Medicine and Toxicology and involved 100 heads of unclaimed adult bodies that underwent post-mortem examination. Detailed morphometric measurements were taken using ImageJ software (National Institutes of Health, Bethesda, MD), focusing on landmarks critical for surgical approaches: the width of the trigeminal ganglion (TG), distances from the medial surface of the posterior root of the zygoma to TG (A), the lateral end of the petrous ridge to TG (B), the arcuate eminence to TG (C), the facial nerve hiatus to TG (D), the foramen spinosum to TG (E), the foramen ovale to TG (F), the superior margin of the internal acoustic meatus to the petrous ridge (G), the lateral end of the petrous ridge to the hiatus for the greater superficial petrosal nerve (GSPN) (H), the foramen spinosum to the foramen ovale (I), and the foramen spinosum to the lateral wall of the middle cranial fossa (J). Data were statistically analyzed using paired sample t-tests and Wilcoxon rank-sum tests.

Results

Significant differences were observed between the left and right sides for several measurements. The width of the TG averaged 13.57 ± 1.53 mm on the left and 14.20 ± 2.09 mm on the right (p = 0.029). Distances from the medial surface of the posterior root of the zygoma to TG (A) were 33.30 ± 6.26 mm (left) and 33.40 ± 5.52 mm (right) (p = 0.001). The lateral end of the petrous ridge to TG (B) measured 37.90 ± 5.72 mm (left) and 40.30 ± 3.35 mm (right) (p = 0.000). Other significant differences included distances from the arcuate eminence to TG (C), which were 28.50 ± 4.20 mm on the left and 29.60 ± 4.31 mm on the right (p = 0.001); the hiatus for the GSPN to TG (D), which were 11.00 ± 1.00 mm on the left and 10.70 ± 0.81 mm on the right (p = 0.003); and the foramen spinosum to TG (E), which were 12.60 ± 2.24 mm on the left and 11.60 ± 1.92 mm on the right (p = 0.001).

Conclusion

In summary, this study presents an in-depth morphometric analysis of important landmarks in the petrous bone, revealing notable differences between the left and right sides. These results emphasize the crucial variability that should be considered when planning surgeries involving the middle and posterior cranial fossa.

## Introduction

The petrous bone, a key structure in the cranial base, delineates the middle and posterior cranial fossae. Its anatomical complexity includes a base, apex, and three surfaces, housing the auditory apparatus internally [1]. Lateral to the apex lies the trigeminal (gasserian) ganglion. Notably, the anterosuperior surface of the petrous bone features arcuate eminence (AE), which overlies the superior semicircular canal [1]. Adjacent to this, a groove directs the greater superficial petrosal nerve (GSPN) toward the foramen lacerum, while further anterolaterally lies another groove for the lesser superficial petrosal nerve, leading towards the foramen ovale (FO) [1].

Near the middle of the posterior surface, the porusacusticus (internal acoustic meatus (IAM)) transmits the VII and VIII cranial nerves alongside labyrinthine vessels [2]. Inferiorly, the carotid canal on the petrous bone's inferior surface runs forward and medially, emerging at the apex to accommodate the internal carotid artery (ICA) [2].

In the medial floor of the middle cranial fossa, the petrous bone interacts closely with the greater wing of the sphenoid bone, hosting crucial neurovascular structures like the FO and foramen spinosum (FS). The superior and inferior petrosal sinuses are also intimately associated with it [3]. Numerous anatomical and radiological studies have documented variations in structures adjacent to the petrous bone, highlighting the critical need for understanding these variations to mitigate intraoperative risks [3-6].

Lateral skull base approaches, including the middle cranial fossa and transpetrosal techniques, are commonly used to address pathologies such as petroclival meningiomas and trigeminal/vestibular schwannomas. Additionally, the retrosigmoid approach provides access to lesions in the cerebellopontine angle, while the osteoplastic craniotomy offers extensive exposure by removing a segment of the skull bone. The endoscopic endonasal approach allows minimally invasive access to the anterior skull base, useful for tumor-like nasopharyngeal lesions. The superior transpetrosal approach enhances access to the anterior petrous bone and its structures. Each approach is selected based on the specific pathology and required exposure, emphasizing the importance of tailored surgical strategies [7]. Otolaryngologists often utilize landmarks on the petrous bone, including the AE, superior semicircular canal, porusacusticus, and foramina such as ovale and spinosum, to access the internal auditory canal (IAC) through drilling procedures [7].

Detailed knowledge of the petrous and peripetrous regions, including awareness of anatomical variations, is crucial for planning and selecting appropriate skull base approaches [8]. This understanding aids neurologists and radiologists in comprehending the anatomical basis of various pathological syndromes and reaching accurate differential diagnoses [8]. Transtemporal surgical approaches facilitate wide exposure of neoplastic, vascular, and traumatic lesions of the cranial base without extensive brain retraction [9].

Previous morphometric analyses of the petrous bone have aimed to quantitatively evaluate the relationships of these bony landmarks critical for skull base approaches [10]. However, there remains a scarcity of literature specifically addressing the Indian population. Therefore, this study aimed to analyze the morphometric relationships between identifiable surface landmarks of the petrous bone and the adjoining middle cranial fossa in Indian adults.

## Materials and methods

Study design

This prospective cross-sectional observational study was conducted from December 2018 to November 2022 by the Department of Anatomy, Atal Bihari Vajpayee Institute of Medical Sciences (ABVIMS) and Dr. Ram Manohar Lohia (RML) Hospital New Delhi. Ethical approval (approval number: 270 (43/2018) IEC/PGIMER/RMLH/4029/18) was obtained from the institute's ethical committee prior to the commencement of the study.

Study participants

The study included 100 heads (200 sides) of unclaimed adult bodies (aged above 18 years) that underwent postmortem examination in the Department of Forensic Medicine and Toxicology, Atal Bihari Vajpayee Institute of Medical Sciences and Dr. Ram Manohar Lohia Hospital, New Delhi. Healthy adults of either sex were included, excluding cases with badly crushed skulls or petrified bodies.

Sample size calculation

The sample size was determined based on the expected prevalence of anatomical variations in the population under study. We aimed to achieve a confidence level of 95% with a margin of error of 5%. Assuming an estimated prevalence of 50% to ensure maximum variability, the calculated sample size was approximately 100 heads (200 sides), which we included in the study. The sample size was calculated using the formula for a single proportion: n=Z2p(1−p)E2, where n is the sample size, Z is the Z-score for a 95% confidence level (1.96), p is the estimated prevalence (0.50), and E is the margin of error (0.05)

Study procedure

**Figure 1:**
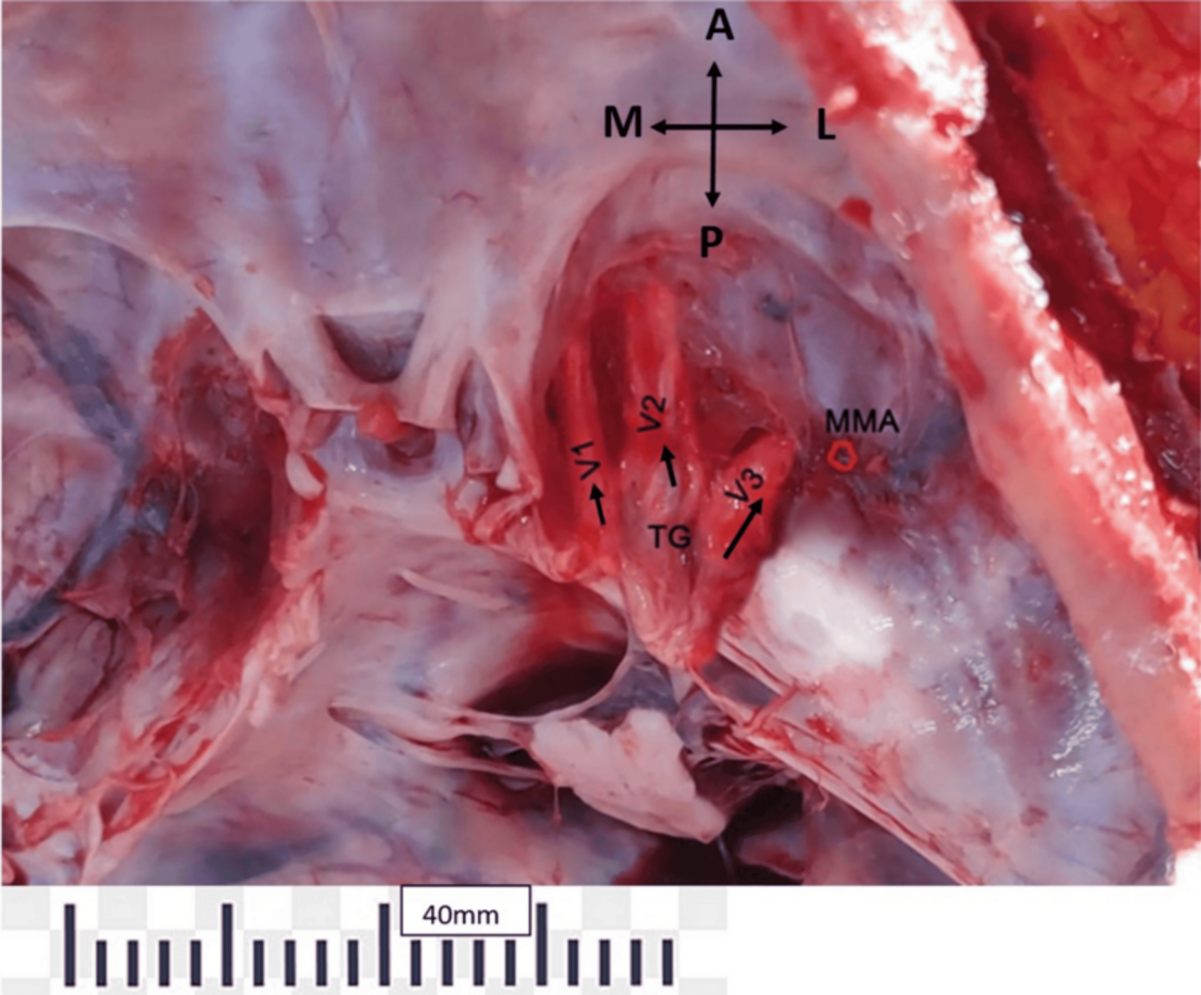
The right temporal lobe has been removed along with duramater to expose the middle cranial fossa. Note the trigeminal ganglion (TG); three divisions of trigeminal; ophthalmic (V1); maxillary (V2); mandibular (V3); and middle-meningeal artery (MMA).

The calvaria was carefully opened, and the brain was removed to preserve the rootlets of cranial nerves entering various foramina. The dura mater covering the middle cranial fossa and posterior cranial fossa was dissected to expose the petrous bone and associated landmarks. Key landmarks for anterior petrosectomy and middle cranial fossa approaches included the AE, the apex of the petrous bone, the medial surface of the root of zygoma, trigeminal ganglion (TG), ophthalmic nerve (V1), maxillary nerve (V2), mandibular nerve (V3), FO, foramen rotundum (FR), GSPN, and FS. On the posterior aspect of the petrous bone, landmarks such as the trigeminal porus (TP) and porus acusticus/IAM (PA/IAM) were considered (Figure 1).

Measurements

**Figure 2:**
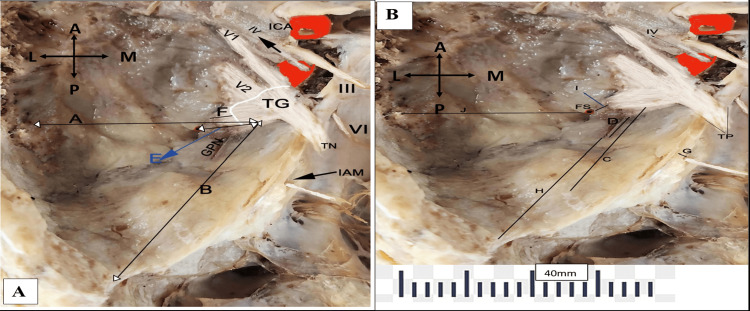
(A, B) Left middle cranial fossa exposed to view the neurovascular bundle and the measurements taken. ICA: Internal carotid artery, IAM: Internal acoustic meatus, TN: Trigeminal nerve, TP: trigeminal porous, GPN: Greater petrosal nerve TG: Trigeminal ganglion, FS: Foramen spinosum with middle meningeal artery, V1 (ophthalmic division), V2 (maxillary division), III (oculomotor nerve), IV (trochlear nerve), VI (abducent nerve), medial surface of posterior root of zygoma-TG (A), lateral end of petrous ridge-lateral margin of TG (B), arcuate eminence at petrous ridge-lateral margin of TG (C), facial nerve hiatus-TG (D), FS-TG (E), foramen ovale-TG (F), superior margin of IAM to petrous ridge (G), lateral end of petrous ridge to posterior end of hiatus for GPN (H), FS-foramen ovale (I), and FS-lateral wall of MCF (J).

Detailed morphometric analysis encompassed comprehensive measurements of critical anatomical landmarks essential for surgical approaches. These included the width of the TG, distances such as from the medial surface of the posterior root of the zygoma to TG (A), from the lateral end of the petrous ridge to the lateral margin of TG (B), and from the AE at the petrous ridge to the lateral margin of TG (C). Additionally, measurements were taken from the facial nerve hiatus to TG (D), and distances from the FS to TG (E), from the FO to TG (F), and from the superior margin of the IAM to the petrous ridge (G) were recorded. Further parameters included distances from the lateral end of the petrous ridge to the posterior end of the hiatus for the GSPN (H), from the FS to the FO (I), and from the FS to the lateral wall of the middle cranial fossa (J). All measurements were taken bilaterally using ImageJ software (National Institutes of Health, Bethesda, MD), with calibration performed using standard measurements. Each measurement was repeated three times for accuracy and consistency (Figures 2A, 2B).

Statistical analysis

Statistical analysis was conducted using IBM SPSS version 26.0 (IBM Corp., Armonk, NY, USA). Data were presented as arithmetic means (standard deviation). Differences between the right and left sides were analyzed using the paired sample t-test and Wilcoxon rank-sum test, with statistical significance set at p < 0.05. Outliers and missing data were managed according to established statistical procedures.

Ethical considerations

The use of unclaimed bodies in research and education is governed by specific legal provisions under the Anatomy Act and guidelines provided by the Medical Council of India. These guidelines permit the utilization of unclaimed bodies for academic and research purposes, provided that all efforts to identify and contact the next of kin have been exhausted and the bodies remain unclaimed for a stipulated period. At ABVIMS and Dr. RML Hospital, all unclaimed bodies are handled with respect and dignity. The Department of Forensic Medicine and Toxicology adheres to protocols that include ensuring that all possible steps are taken to identify the deceased, including public notifications and attempts to contact any known relatives. Legal compliance involves observing the mandatory waiting period as prescribed by law before a body is declared unclaimed and eligible for research use. Ethical approval is obtained from the institute's ethical committee to ensure that the study complies with ethical standards and respects the dignity of the deceased. Furthermore, detailed records of all procedures are maintained, ensuring transparency and adherence to institutional and legal requirements. By following these guidelines, the study was conducted with the highest ethical standards, ensuring that all research activities were carried out with sensitivity and respect for the unclaimed individuals.

## Results

The morphometric analysis revealed several significant differences between the left and right sides of the petrous bone. The width of the TG ranged from 10.00 to 16.2 mm on the left side, with a mean ± SD of 13.57 ± 1.53 mm, and from 11.60 to 17.9 mm on the right side, with a mean ± SD of 14.20 ± 2.09 mm (p = 0.029). The distance from the medial surface of the posterior root of the zygoma to TG (A) ranged from 20.40 to 41.30 mm on the left side (mean ± SD: 33.30 ± 6.26 mm) and from 2.09 to 4.06 mm on the right side (mean ± SD: 33.40 ± 5.52 mm) (p = 0.001) (Table 1).

**Table 1:** Morphometric analysis of landmarks in MCF and on the anterior and posterior surface of the petrous bone. *p-value statistically significant TG - trigeminal ganglion, GSPN - greater superficial petrosal nerve, IAM - internal acoustic meatus, MCF - middle cranial fossa

Anatomical landmarks	Left side (mm)		Right side (mm)		P-value
	Range	Mean ±SD	Range	Mean ±SD	
Width of TG	10.00-16.2	13.57±1.53	11.60-17.9	14.20±2.09	0.029*
Medial surface of posterior root of zygoma-TG (A)	20.40-41.30	33.30±6.26	2.09-4.06	33.40±5.52	0.001*
Lateral end of petrous ridge-lateral margin of TG (B)	31.20-51.90	37.90±5.72	27.50-45.60	40.30±3.35	0.000*
Arcuate eminence-lateral margin of TG (C)	15.60-34.40	28.50±4.20	12.70-35.50	29.60±4.31	0.001*
GSPN hiatus-TG (D)	11.00 ± 1.00	8.20-12.40	7.40-11.80	10.70 ±0.81	0.003*
Foramen spinosum-TG (E)	8.82-15.60	12.60±2.24	7.81-18.50	11.60±1.92	0.001*
Foramen ovale-TG (F)	4.51-9.01	7.10±1.22	4.80-9.21	7.10±1.25	0.040*
Superior margin of IAM to petrous ridge (G)	1.60-2.50	2.12±0.31	1.20-2.40	2.04±0.34	0.080
Lateral end of petrous ridge to posterior end of hiatus for GSPN (H)	30.20-33.60	31.78±1.31	32.00-34.50	32.30 ±1.16	0.001*
Foramen spinosum-foramen ovale (I)	2.01-3.10	2.69±0.30	2.00-3.00	2.56±0.35	0.001*
Foramen spinosum-lateral wall of MCF (J)	20.02-27.50	24.91±1.83	21.20-28.6	24.86±1.86	0.070

The distance from the lateral end of the petrous ridge to the lateral margin of TG (B) ranged from 31.20 to 51.90 mm on the left side (mean ± SD: 37.90 ± 5.72 mm) and from 27.50 to 45.60 mm on the right side (mean ± SD: 40.30 ± 3.35 mm) (p < 0.001). The distance from the AE to the lateral margin of TG (C) ranged from 15.60 to 34.40 mm on the left side (mean ± SD: 28.50 ± 4.20 mm) and from 12.70 to 35.50 mm on the right side (mean ± SD: 29.60 ± 4.31 mm) (p = 0.001) (Table 1).

The distance from the hiatus for the GSPN to TG (D) showed a significant difference, with a mean ± SD of 11.00 ± 1.00 mm on the left side and 10.70 ± 0.81 mm on the right side (p = 0.003). The distance from the FS to TG (E) ranged from 8.82 to 15.60 mm on the left side (mean ± SD: 12.60 ± 2.24 mm) and from 7.81 to 18.50 mm on the right side (mean ± SD: 11.60 ± 1.92 mm) (p = 0.001) (Table 1).

Other notable differences included the distance from the FO to TG (F), which ranged from 4.51 to 9.01 mm on the left side (mean ± SD: 7.10 ± 1.22 mm) and from 4.80 to 9.21 mm on the right side (mean ± SD: 7.10 ± 1.25 mm) (p = 0.040). The distance from the superior margin of the IAM to the petrous ridge (G) did not show significant variation, with p = 0.080. The distance from the lateral end of the petrous ridge to the posterior end of the hiatus for GSPN (H) was significantly different, with a mean ± SD of 31.78 ± 1.31 mm on the left side and 32.30 ± 1.16 mm on the right side (p = 0.001) (Table 1).

Additionally, the distance from the FS to the FO (I) ranged from 2.01 to 3.10 mm on the left side (mean ± SD: 2.69 ± 0.30 mm) and from 2.00 to 3.00 mm on the right side (mean ± SD: 2.56 ± 0.35 mm) (p = 0.001). The distance from the FS to the lateral wall of the middle cranial fossa (J) did not show significant variation, with p = 0.070 (Table 1).

## Discussion

This study presents a comprehensive morphometric analysis of critical anatomical landmarks within the petrous bone, emphasizing asymmetries and their implications for surgical approaches in the middle and posterior cranial fossa. The findings highlight the intricate variability in dimensions between the left and right sides, offering valuable insights into anatomical considerations essential for neurosurgical planning.

Comparative analysis with previous studies reveals consistent observations regarding asymmetries in petrous bone dimensions. Our findings align closely with studies by Prakash et al. and Zhang et al., which similarly highlight the variability in cranial nerve rootlet positions and their pivotal role in surgical strategy [11,12]. The width of the TG in our study revealed significant side-to-side differences, measuring 13.57 ± 1.53 mm on the left and 14.20 ± 2.09 mm on the right (p = 0.029). In comparison, Ogut et al. reported dimensions for the TP, with an average height (HD) of 8.02 mm (female) and 9.2 mm (male) on the right side, and 8.26 mm (female) and 8.81 mm (male) on the left side. The vertical diameter (VD) of the TP in their study averaged 1.99 mm (female) and 2.65 mm (male) on the right side, and 2.42 mm (female) and 2.94 mm (male) on the left side [13]. These comparisons highlight notable variations between the TG width and TP dimensions, emphasizing the importance of considering anatomical differences in surgical planning. The differences observed in TG width align with broader anatomical variability reported in recent literature, reinforcing the need for tailored surgical approaches based on individual anatomical characteristics.

This variation underscores the necessity for individualized surgical approaches, which are crucial for procedures such as vestibular schwannoma resection and petroclival meningioma excision. In addition to the middle cranial fossa and transpetrosal approaches, which are commonly used for these pathologies, other significant surgical approaches include the retrosigmoid approach, which provides access to the cerebellopontine angle and is effective for managing tumors like acoustic neuromas. The osteoplastic craniotomy offers extensive exposure by removing a section of the skull, allowing for comprehensive access to large lesions. The endoscopic endonasal approach has also emerged as a minimally invasive option for addressing anterior skull base tumors, providing direct access without extensive retraction. Each of these approaches has its own advantages and is selected based on the specific characteristics of the lesion and the anatomical variations of the patient. A thorough understanding of these approaches and their indications is essential for optimizing surgical outcomes and minimizing risks [14,15].

Measurements from the medial surface of the posterior root of the zygoma to the TG in our study revealed precise asymmetries, with measurements of 33.30 ± 6.26 mm on the left and 33.40 ± 5.52 mm on the right (p = 0.001). This finding is particularly relevant when compared with the study by Sekerci et al., which reported that elliptical (or transverse) and oval (or round) porus acusticus internus (PAI) was identified in Turkish dry temporal bones. Specifically, 35.8% of PAIs were elliptical and 64.1% were oval, with a higher prevalence of round PAIs on both sides (32; 26.6% on the left and 39; 32.5% on the right) (p < 0.05). Our study indicates a similarity between the Indian and Turkish dry temporal bones regarding the distance from the PAI to the superior semicircular plane (SSP) and the superior semicircular sulcus (SSS), which was 7-8 mm and 19-20 mm, respectively (p = 0.01). Additionally, the prevalence of PAI morphology (oval and elliptical) was comparable between the two populations (p = 0.04, p < 0.05). These findings highlight that despite regional anatomical variations, certain morphometric characteristics of the petrous bone and its landmarks are consistent across different populations, which can influence surgical planning and approach [16]. These comparative observations underscore the variability in cranial base anatomy and highlight the importance of individualized surgical planning. These findings resonate with research by Chaware et al., emphasizing the critical role of accurate anatomical knowledge in minimizing surgical risks associated with cranial nerve manipulation [17].

Furthermore, our study's focus on distances from the lateral end of the petrous ridge to the lateral margin of TG (B) elucidates essential anatomical landmarks necessary for safe surgical navigation (3.79 ± 0.57 mm on the left vs. 4.03 ± 0.35 mm on the right, p < 0.001). These observations are consistent with studies by Tayebi et al., highlighting the variability clinicians must consider when accessing intricate structures like the internal auditory canal and jugular foramen [18].

The distance from the AE to the lateral margin of the TG (C) showed significant variation between sides, and this variation is clinically relevant, as the AE is a crucial landmark in middle cranial fossa approaches. Its proximity to the TG underscores the need for precise localization to avoid nerve damage during surgical interventions. Comparable findings were noted by Arslan et al. and Singh et al., who emphasized the variability of the AE in different populations [19,20].

The distance from the FS to the TG in our study averaged 12.60 ± 2.24 mm on the left and 11.60 ± 1.92 mm on the right (p = 0.001). This asymmetry is crucial for surgical approaches to the middle cranial fossa, where precise identification of the FS is essential to avoid damaging vascular and neural structures during procedures such as tumor resection. Abdelghani et al. found that the optic canal (OC) depth, IAM width, and the diameters of cranial nerves VII and IX were significantly greater on the left side, while cranial nerve VI length, nerve V diameter, and the lengths of cranial nerves XI and the distances of the hypoglossal canal (HC) and accessory HC from the skull were significantly greater on the right side (p < 0.05) [21]. These findings align with those of Zdilla et al., who also noted the significance of the FS in neurosurgical procedures [22].

The measurement from the FO to the TG in our study averaged 7.10 ± 1.22 mm on the left and 7.10 ± 1.25 mm on the right (p = 0.04). This distance is critical for surgical procedures targeting the TG, such as those aimed at treating trigeminal neuralgia, where precise localization of the FO helps minimize the risk of neural damage. This finding is consistent with the study by Poornima et al., which reported average transverse and anteroposterior diameters of the FO as 3.50 ± 0.84 mm and 6.4 ± 1.47 mm on the left side, and 3.54 ± 0.57 mm and 6.5 ± 1.40 mm on the right side. Poornima et al. also observed various shapes of the FO, including oval (60%), almond (25%), round (13%), and slit-like (2%) foramina. Additionally, 11% of foramina had spines, 5% had tubercles, and 10% had a bony plate, with duplication noted in one skull on the right side [23].

The measurement between the FS and the FO (I) in our study revealed significant variability, highlighting the importance of careful preoperative planning to avoid nerve damage during surgical procedures in the middle cranial fossa. This variability aligns with observations by Naqshi et al., who reported a confluent FO and FS in one skull on the left side, indicating potential anatomical variations that can impact surgical navigation. Additionally, Naqshi et al. documented a duplication of the FS in one skull on the right side and an absence of the FS in another skull on the right side [24]. John et al. also noted the importance of this measurement for safe surgical access to the middle cranial fossa [25].

The clinical implications of these findings are significant, providing valuable insights for optimizing surgical outcomes and minimizing intraoperative risks associated with anatomical variations. The precise measurements and detailed anatomical nuances highlighted in our study are crucial for refining surgical approaches to the middle cranial fossa. For instance, understanding the variability in distances between key landmarks such as the FS, FO, and the TG helps tailor surgical planning and approach. This includes selecting appropriate techniques - such as the middle cranial fossa approach, transpetrosal approach, or retrosigmoid approach - to ensure effective and safe management of conditions like trigeminal neuralgia, vestibular schwannomas, and petroclival meningiomas. By delineating these anatomical differences, our study aids in advancing cranial base surgery toward more precise and personalized patient care strategies, enhancing the accuracy of surgical interventions and improving overall patient outcomes [26].

Limitations

While this study provides valuable insights into the morphometric variations of the petrous bone, several limitations should be acknowledged. Firstly, the sample size, although adequate for morphometric analysis, consisted solely of unclaimed cadaveric heads, potentially limiting the generalizability to living populations. Secondly, despite efforts to standardize measurements using ImageJ software, inherent anatomical variations and post-mortem changes may have influenced accuracy. Thirdly, the study's cross-sectional design precludes establishing causal relationships or capturing longitudinal changes over time. Additionally, while statistical analyses were robust, variations in measurement techniques or inter-observer variability were not fully addressed. Also, due to the nature of our study, which involved unclaimed adult bodies, detailed demographic information such as the exact ratio and percentage of males and females was not available. Lastly, the exclusion of specific pathological conditions or demographic factors, such as age-related changes, may limit the comprehensive understanding of anatomical variability in broader clinical contexts. Future studies addressing these limitations could provide further insights into the clinical relevance of petrous bone morphology.

## Conclusions

In conclusion, this study offers a detailed morphometric analysis of key petrous bone landmarks, revealing significant left-right asymmetries. Compared to previous research, our findings provide additional insights into the precise distances between landmarks like the FS and FO. These results highlight both consistencies and differences with earlier studies, emphasizing the need for individualized surgical planning. By clarifying precise anatomical measurements and their implications, our research strengthens the basis for safer and more effective neurosurgical procedures, particularly when facing complex conditions such as vestibular schwannomas and petroclival meningiomas. We believe that incorporating these insights into clinical practice will enhance surgical approaches and benefit patients by reducing risks associated with anatomical variations.
